# Preclinical studies of low back pain

**DOI:** 10.1186/1744-8069-9-17

**Published:** 2013-03-28

**Authors:** Judith A Strong, Wenrui Xie, Feguens J Bataille, Jun-Ming Zhang

**Affiliations:** 1 Pain Research Center, Department of Anesthesiology, University of Cincinnati College of Medicine, Cincinnati, OH 45267-0531, USA

**Keywords:** Sensory ganglia, Spontaneous activity, TNF-α, MCP-1, IL1-β, Cxcl1, Eplerenone, Mineralocorticoid receptor, Glucocorticoid receptor

## Abstract

Chronic low back pain is a major cause of disability and health care costs. Current treatments are inadequate for many patients. A number of preclinical models have been developed that attempt to mimic aspects of clinical conditions that contribute to low back pain. These involve application of nucleus pulposus material near the lumbar dorsal root ganglia (DRG), chronic compression of the DRG, or localized inflammation of the DRG. These models, which are primarily implemented in rats, have many common features including behavioral hypersensitivity of the hindpaw, enhanced excitability and spontaneous activity of sensory neurons, and locally elevated levels of inflammatory mediators including cytokines. Clinically, epidural injection of steroids (glucocorticoids) is commonly used when more conservative treatments fail, but clinical trials evaluating these treatments have yielded mixed results. There are relatively few preclinical studies of steroid effects in low back pain models. One preclinical study suggests that the mineralocorticoid receptor, also present in the DRG, may have pro-inflammatory effects that oppose the activation of the glucocorticoid receptor. Although the glucocorticoid receptor is the target of anti-inflammatory steroids, many clinically used steroids activate both receptors. This could be one explanation for the limited effects of epidural steroids in some patients. Additional preclinical research is needed to address other possible reasons for limited efficacy of steroids, such as central sensitization or presence of an ongoing inflammatory stimulus in some forms of low back pain.

## Review

### Preclinical models of low back pain

Low back pain is a common ailment which virtually everyone experiences at some point. In 30% of cases low back pain becomes chronic. This is a major cause of disability and an important driver of health care costs in the United States and other countries. With current therapies many patients fail to achieve adequate relief for chronic pain conditions including chronic low back pain. Causes of low back pain can include problems with the lumbar intervertebral discs, such as disc herniation, displacement or degeneration; compression of nerve roots as may occur with spinal stenosis; and inflammatory conditions such as arthritis. In the majority of cases of chronic low back pain the etiology is unknown, however [1-5].

**Figure 1:**
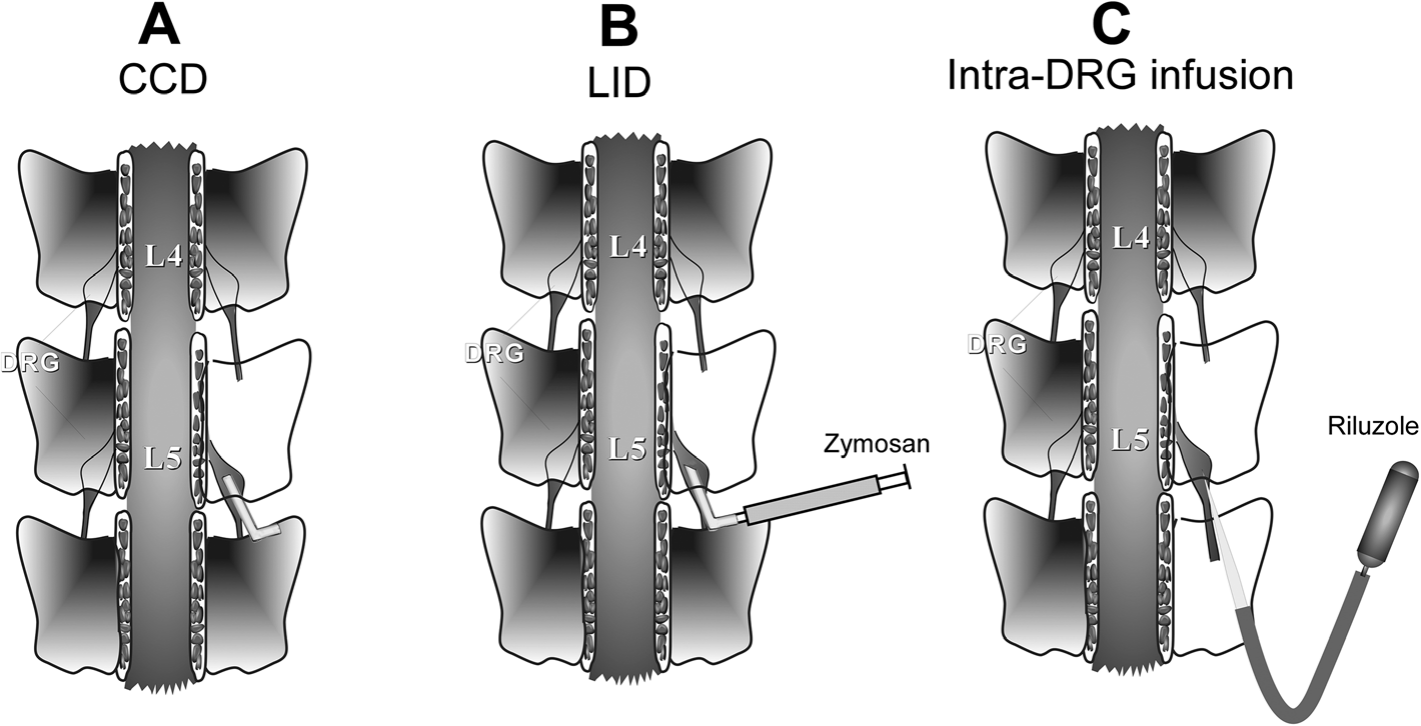
**Diagram of some techniques used in rat models of low back pain. A**: in the chronic compression of the dorsal root ganglion (CCD) model, a metal rod is inserted over the lumbar DRG and left in place. **B**: LID: local inflammation of the dorsal root ganglion is achieved by depositing the immune stimulator zymosan in incomplete Freund’s adjuvant over the lumbar DRG. **C**: Continuous local application of drugs such as the persistent Na current blocker riluzole to the DRG is achieved by connecting an osmotic pump to a small glass needle, inserted beneath the perineurium of the adjacent spinal nerve.

A number of preclinical models have been developed that attempt to mimic some of the above known causes of low back pain. Because a ruptured disc can expose the nearby dorsal root ganglion and its nerve roots to nucleus pulposus (NP), an early model of low back pain involved applying NP to the lumbar dorsal root ganglia (DRG) and/or adjacent nerve roots. This was implemented in pigs [6] and later in rats [7]. Several variations of rat NP models are described in the literature, differing in the source of the NP (harvested from a tail disc or from a disc adjacent to the lumbar DRG to be treated, with or without intervening time), the exact location in which the NP is applied (spinal roots, DRG, cauda equina), and the inclusion of additional maneuvers such as root compression, pinch, or brief DRG displacement. Another commonly used rat model is the chronic compression of the DRG model (CCD), in which one or two lumbar DRGs are compressed by inserting small L-shaped metal rods beneath the intervertebral foramen, which remain in place for the duration of the experiment [8,9] (Figure 1). A variant of this model in which the compression is achieved with a hemostatic matrix (SURGIFLO) instead of a metal rod has also been described [10]. The CCD model has also been implemented in mice [11]. In another rat model, the L5 DRG is inflamed locally by depositing over it a small drop of the immune stimulator zymosan in incomplete Freund’s adjuvant [12]. Inflammatory processes are thought to play key roles in low back pain (see below) as well as contributing to neuropathic pain conditions [13]. Hence the rationale for developing this model was that it allows the direct effects of inflammation on the sensory neurons to be studied in the absence of overt axotomy. Other approaches to locally inflaming the lumbar DRG region use complete Freund’s adjuvant (CFA) [14] and injection of inflammatory soup into the intervertebral foramen [15].

### Pain behaviors in rodent low back pain models

All of the above types of back pain models implemented in rodents show increased mechanical sensitivity in the ipsilateral hind paw as measured with von Frey filaments, and many also show increased sensitivity of the paw to hot or cold thermal stimuli. The duration of this hypersensitivity varies considerably between models but can be very long-lasting; for example, local inflammation of the DRG with zymosan (LID) caused mechanical hypersensitivity lasting over 40 days [16].

**Figure 2:**
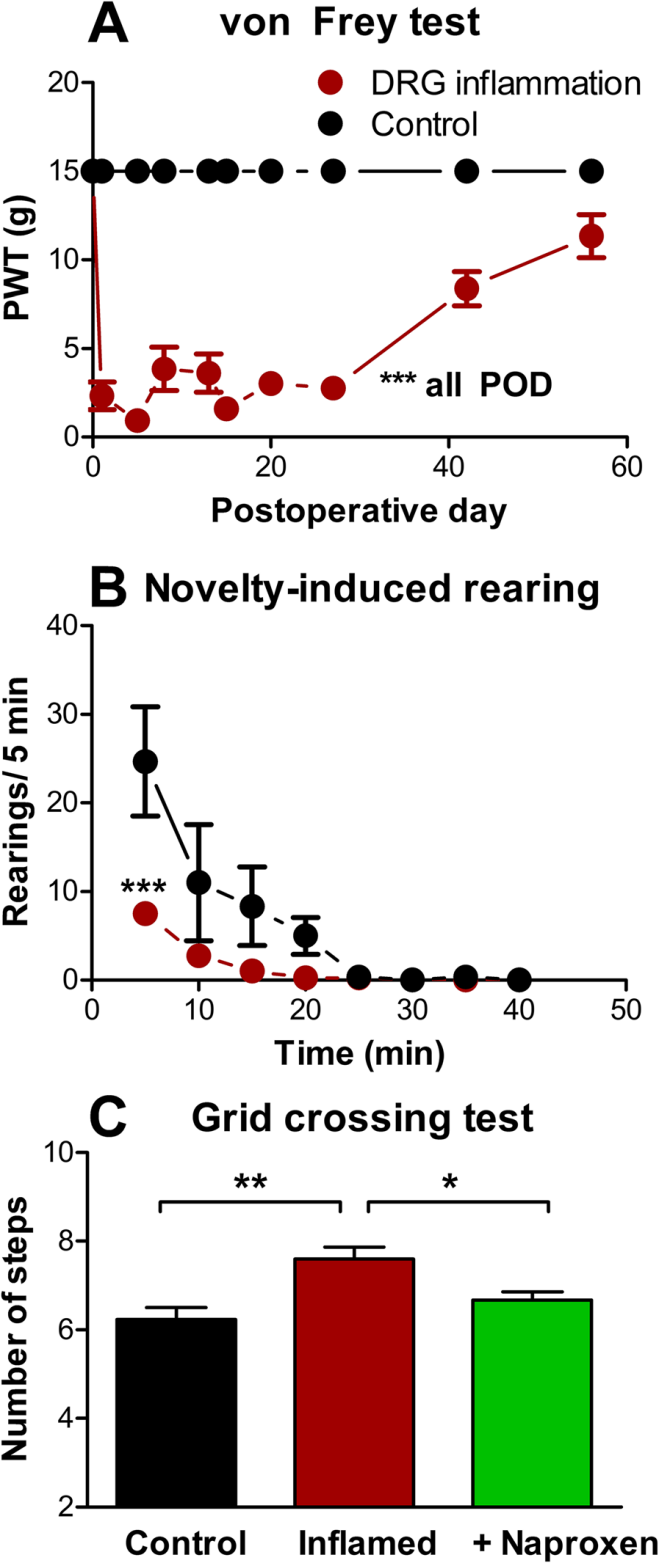
**Alternative behavior measures in a low back pain model. A**: Inflammation of the L5 DRG (LID model) resulted in prolonged hypersensitivity of the ipsilateral paw as measured with the von Frey test; control animals all failed to respond to the maximum stimulus applied throughout the testing period. **B**: reduction in rearing behavior when the rat was placed in a novel chamber, measured 3 days after DRG inflammation. Data in A and B adapted from reference [16]. **C**: rats (n = 6) took a larger number of shorter steps to cross a wire grid after DRG inflammation, measured 1 day after DRG inflammation, an effect that was reversed by oral naproxen (30 mg/kg given 2 hours prior to testing). *, p < 0.05, **, p < 0.01, ***, p < 0.001 significant difference between the indicated values (ANOVA).

Some recent studies have called into question the relevance of reflexive measures such as the von Frey test or thermal threshold tests to understanding clinical chronic pain states [17]. Regarding models of low back pain, an interesting commentary by Devor and Tal [18] notes that mechanical allodynia in the foot is apparently not commonly observed in human low back pain patients, and questions the degree to which animal models relying on hindpaw reflex tests are truly modeling low back pain or, in particular, sciatica. As for other types of pain models, some recent studies of low back pain models have tried to use more complex measures of pain behavior. In the LID model, it has been demonstrated that local DRG inflammation reduces the rearing behavior (but not horizontal locomotion) observed after the rat is placed in a novel environment. This effect could be reversed by naproxen, which had no effect on rearing in normal animals [16]. In the same model, an increase in the number of steps used to cross a metal grid (i.e., shorter steps were taken) has also been observed (Figure 2; unpublished results from our laboratory). This was also reversed by naproxen. Both these observations are consistent with the idea that rats are avoiding stretching the lower back region after DRG inflammation. In a model in which NP is applied to the L5 DRG on one side, changes in gait and weight bearing were observed. Gait was asymmetrical (suggesting a limp) and weight was shifted to the contralateral limb [19]. Limping could also be observed in some animals subjected to light lumbar DRG compression plus exposure of the DRG to NP from a nearby experimentally ruptured disc [20]. In a mouse model (mice genetically null for a matricellular protein found in intervertebral discs) in which spontaneous disk degeneration is observed with age, several different tests were implemented to test the hypothesis that degenerating discs caused back pain [21]. The mutant mice showed impaired grip force (a test involving stretching of the back), and in a test in which they were suspended by the tail showed more time in behaviors that avoided the gravity-induced stretching of the spine. In addition, exploratory behavior in mutant but not wildtype mice could be reduced by a preceding tail suspension, and activity in a maze that required flexing of the back was reduced. It would be interesting to see if these tests, which seem to have greater face validity for low back pain than some of the more commonly used behavioral tests involving hind paw reflexes, could be adapted for use in rat models of low back pain. Mutant mice also showed significantly higher cold sensitivity in the hindpaw as they aged. This suggests that sensitivity measured in a reflexive test applied to the rodent hindpaw can correlate with more direct low back pain measures. It may be that tests applied to the hindpaw in rodent models are relevant to low back pain even though they have no counterpart in humans, because the relatively smaller and less well-defined map of the hindquarter region in rats compared to humans may allow greater spread of pain stimuli affecting the lower back into the regions innervating the hindpaw [18]. Differences between species that walk on four vs. two legs may also contribute to the hindpaw hypersensitivity observed in rodent models after manipulating the lumbar DRG.

### Neuronal changes in low back pain models

Several studies have examined changes in properties of the sensory neurons in compressed (CCD model) or inflamed (LID model) sensory ganglia. These two models apparently have many effects in common, including decreased rheobase and increased firing in response to injected current, as well as increased spontaneous activity or evoked bursting [8,12,22]. Such changes can also be observed in a model in which pain behaviors are induced by injecting an inflammatory “soup” onto the lumbar DRG [15]. Although increased excitability was observed in all neuron types (based on diameter or conduction velocity) in these studies, the increase in spontaneous activity or evoked bursting was observed primarily in large and medium diameter neurons or those with Aαβ conduction velocities. In all size classes, at least some of the excitability changes are intrinsic to the sensory neurons as they are preserved in short term culture [23,24]. Many studies examining the possible ionic basis of this increased excitability and spontaneous activity have focused on voltage-gated sodium channels. These channels can mediate persistent sodium currents that may contribute to spontaneous firing. The CCD model caused increased persistent Na currents in medium and large diameter neurons [25]. It is noteworthy that gabapentin, which is commonly used for chronic pain, blocked persistent Na currents in medium diameter DRG neurons from CCD ganglia at clinically relevant concentrations, which are lower than the concentrations required to affect the K and Ca channels that are usually considered to be the therapeutic target of this drug [26]. Gabapentin also reduced mechanical hypersensitivity in the LID model [16]. The importance of this spontaneous activity in myelinated neurons is suggested by a study in which riluzole applied to the inflamed DRG in vivo during the first 7 days greatly reduced mechanical hypersensitivity. Riluzole shows some selectivity for persistent Na currents over transient Na currents; in the same study, spontaneous activity of myelinated neurons from locally inflamed DRG was more sensitive to riluzole than were evoked action potentials [16]. Riluzole also blocks spontaneous activity in medium and large diameter fibers induced by the CCD model [25]. An even stronger correlation between spontaneous activity and pain behaviors was observed in a study in which in vivo knockdown of the Na_v_1.6 sodium channel isoform at the time of local DRG inflammation completely blocked both mechanical hypersensitivity and abnormal spontaneous activity in myelinated cells, while having minimal effects on normal action potentials or behavior in normal animals [27]. This channel was chosen for study because it can mediate both persistent and resurgent Na currents; the latter can allow the type of high-frequency firing that is observed in spontaneously active cells in this model.

Most nociceptors are small diameter, unmyelinated or thinly myelinated cells. It is not yet understood how spontaneous activity, which is predominantly observed in myelinated cells, could contribute to pain behaviors in these low back pain models. One possibility is that at least some of the spontaneously active cells are myelinated nociceptors. In rat, 20% of Aαβ cells have nociceptive thresholds and response properties, and project into lamina I or II of the dorsal horn [28,29]. Other possibilities, as discussed in the excellent review by Devor [30], include phenotypic switching by Aαβ cells that begin to express nociceptive transmitters or activity-dependent changes in processing of normally innocuous stimuli by the spinal cord. Most research on such mechanisms has focused on neuropathic pain models and it would be of interest to conduct such studies in low back pain models.

### Role of inflammation

Inflammation has long been assumed to be a component of low back pain, which is often at least partially relieved by systemic nonsteroidal anti-inflammatory drugs or local injections of glucocorticoids (see below). In the low back pain rodent models discussed above, signs of inflammatory response in the DRG have been observed, such as activation of satellite glia cells, infiltration of macrophages, elevation of pro-inflammatory cytokines, and activation of inflammatory signaling pathways [7,12,14,31-33]. The NP from a ruptured disc may not only compress the nearby nerve roots or DRG, but can also serve as a stimulus for inflammation (since this material is normally isolated from the immune system, it acts like a foreign protein), and was also found to be itself a source of pro-inflammatory cytokines. Much work has focused on the cytokines interleukin-1β (IL-1β) and tumor necrosis factor α (TNF-α), both of which are found in NP. These cytokines can induce pain behaviors when applied to the lumbar DRG or nerve roots, and interfering with TNF-α reduces pain behaviors in both NP-based models and the CCD model [32,34-36]. However, a recent randomized clinical trial of the TNF-α antagonist etanercept in radiculopathy patients failed to show positive effects [37]. It has been suggested in some preclinical studies that TNF-α is important only during the early stages of the models [35,38,39]. This may account for some clinical failures of TNF-α antagonism since such trials are likely to involve patients in which low back pain has become chronic. Another pro-inflammatory cytokine that has received attention is monocyte chemoattractant protein 1 (MCP-1; systemic name Ccl2). MCP-1 is elevated in CCD and LID models [12,40], and has direct excitatory effects on sensory neurons [40,41]. The cytokine growth-related oncogene 1 (GRO/KC; systemic name Cxcl1) is also elevated in both LID and NP models [12,32]. This cytokine, analogous to interleukin-8 in humans, is best known for its role in recruiting neutrophils. However, GRO/KC receptors are expressed on neurons, and GRO/KC incubation has direct excitatory effects on small diameter DRG neurons in primary culture [42]. More generally, DRG neurons express receptors for several pro-inflammatory cytokines, and these cytokines usually have excitatory effects when applied to neurons in the ex vivo isolated whole DRG preparation or to acutely cultured neurons [43,44]. In the CCD model, DRG neurons show increased sensitivity to an inflammatory soup or to MCP-1 when compared to neurons from naïve animals [40,45]. Taken together these studies indicate that several pro-inflammatory cytokines locally elevated in the DRG may contribute to low back pain, at least in part through their direct effects on neurons.

The cytokines studied to date were for the most part selected on the basis of prior literature-based knowledge of individual cytokines. With the advent of techniques such as microarrays that examine essentially the entire set of genes in a rat or mouse, it is possible to examine gene expression in low back pain models without any such prior assumptions. A recent microarray study [46] of gene expression in the LID model (samples taken 3 days after DRG inflammation) showed that the model induced large-scale regulation of gene expression (23% of all observed transcripts were significantly regulated). Of these, immune-related genes were the most commonly regulated class. However, many of the previously studied cytokines mentioned above (e.g., TNF-α, IL-1β, GRO/KC) were not significantly regulated at the mRNA level at this time point in the LID model, while other pro-inflammatory cytokines with little previous relationship to pain in the literature were shown to be strongly upregulated at both the day 3 time point used for the microarray, and at a later time point (day 14). Most of these previously unstudied cytokines were in the CXC cytokine family, and some (Cxcl13, Cxcl14, Cxcl15) had been observed in several other microarray studies of DRG gene expression in pain models, suggesting a more general role beyond this particular back pain model.

Although pain models are often classified as neuropathic or inflammatory, some experimental evidence suggests that inflammation occurring locally near the DRG has effects on the sensory neurons which are similar to those observed in neuropathic models involving axotomy near the DRG, but distinct from effects of more distant, peripheral inflammation or more distant axotomy. For example, the gene expression changes induced by local DRG inflammation in the above mentioned microarray study showed the largest overlap with those reported in microarray studies of the spinal nerve ligation model, which is commonly classified as a neuropathic pain model but which involves an injury close to the DRG; there was much less overlap with models involving more distant axotomy. Conversely, there was virtually no overlap with a microarray study of DRG gene expression changes induced by peripheral inflammation (CFA paw injection) [47], and the few overlapping genes were regulated in the opposite direction. In another study, inflammation of the DRG by local application of CFA near the DRG increased COX-2 levels in neurons, but CFA placed distally, either near the sciatic nerve or in the paw, failed to do so [14]. This suggests that some findings in studies with experimentally induced peripheral inflammation, for example, preclinical models of arthritis, may not necessarily apply to low back pain in which the inflammation occurs at the DRG level.

### Preclinical and clinical studies of anti-inflammatory drugs

Reduction of pain behaviors by non-steroidal anti-inflammatory drugs has been demonstrated in the various rodent back pain models. Some studies used systemic application [14,16], but local or epidural effects have also been demonstrated [33,48]. Steroidal anti-inflammatory drugs are commonly used in various low back pain conditions clinically, via local injection into the epidural space (via intraforaminal, caudal, or interlaminar routes). There are few preclinical studies directly examining behavioral effects of glucocorticoids delivered in or near the DRG, perhaps due to the difficulty in gaining access to the DRG in mice or rats. In two rat studies using a CCD model, the clinically used glucocorticoid triamcinolone applied epidurally reduced pain behaviors when given 3 days after the compression began [10,49]. However, different results were obtained when applying drugs at day 10; at this later time point, triamcinolone failed to improve pain behaviors while a glucocorticoid *antagonist* improved them [49].

**Figure 3:**
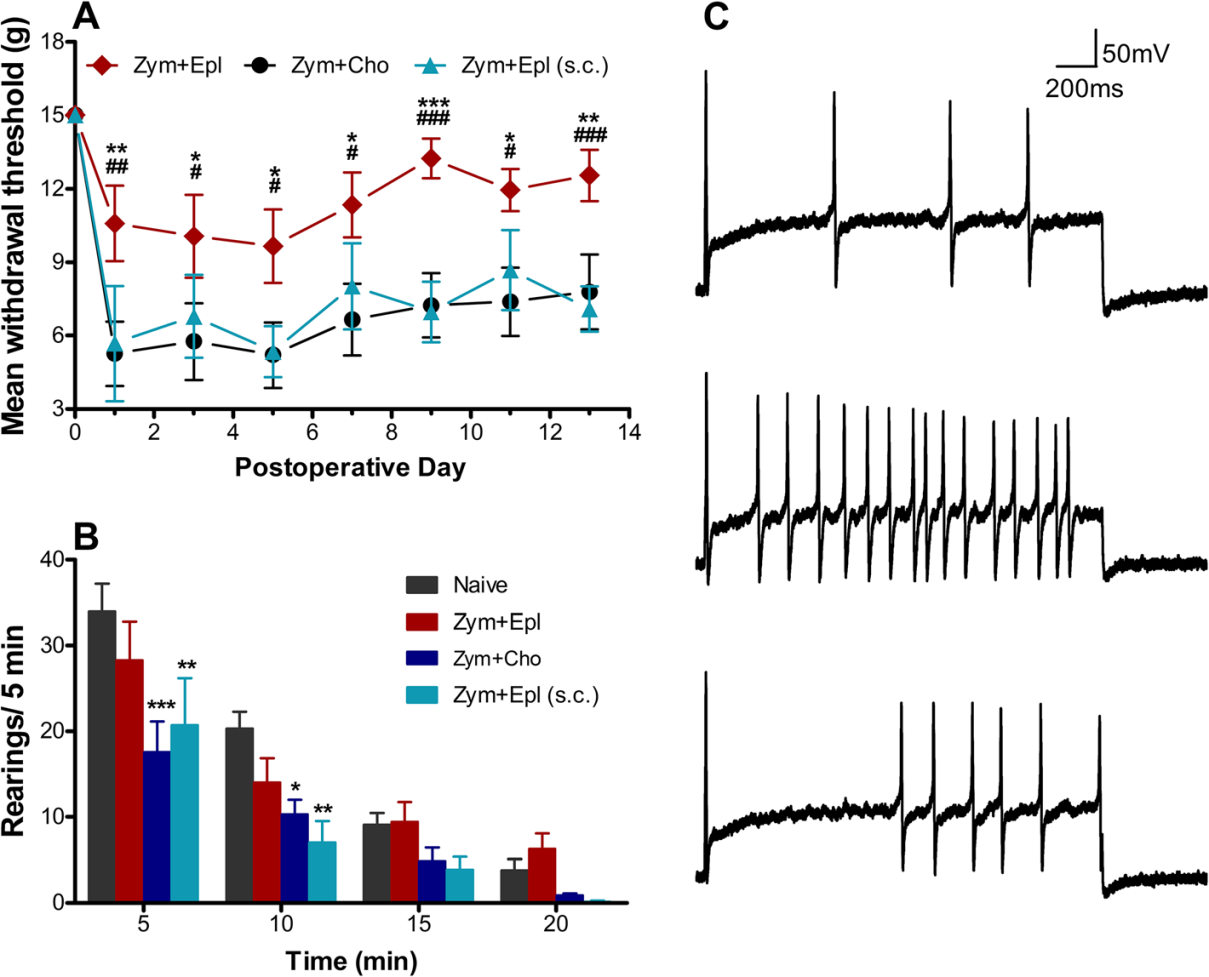
**Effects of a mineralocorticoid antagonist in a DRG inflammation model. A**. Inflammation of the L5 DRG with zymosan/incomplete Freund’s adjuvant (“Zym”) on postoperative day 0 gave a rapid increase in paw withdrawal threshold (PWT) measured with the von Frey method. This was significantly less in animals in which the mineralocorticoid antagonist eplerenone (EPL) was applied locally to the DRG over the same time period, compared to cholesterol (“cho”; chemically inactive control for EPL). * (p < 0.05), ** (p < 0.01), *** (p < 0.001), significant difference between the local EPL and control groups on the indicated day. Systemic EPL, applied subcutaneously (s.c.) did not have the same effect as local EPL. # (p < 0.05), ## (p < 0.01), ### (p < 0.001), significant difference between the local EPL and systemic EPL groups on the indicated day. **B**. rearing behavior in the same 3 experimental groups plus an unoperated group “Naïve”, measured on day 1. Local EPL reversed the inflammation-induced reduction in rearing observed early after the rats were placed in a novel chamber. **C**. Excitability of small isolated DRG neurons measured in vitro measured as number of action potentials fired in response to depolarizing currents was significantly increased in neurons isolated from one day after DRG inflammation (middle trace) compared to neurons isolated from normal DRG (top trace); this effect could be reversed by in vitro EPL application (bottom trace). Adapted from reference [53].

Steroidal anti-inflammatory drugs are agonists of the glucocorticoid receptor (GR), a receptor with widespread tissue distribution which when activated has general anti-inflammatory effects, inhibiting type I inflammation (characterized by high levels of oxidative metabolites and pro-inflammatory cytokines, tissue destruction) while promoting type II inflammation (tissue remodeling and wound repair). However, some clinically used steroids will also activate the mineralocorticoid receptor (MR) in vitro [50,51]. Though best known for its sodium-reabsorbing role in the kidney, more recently the MR has been found to be expressed in other tissues, where its activation may promote type I inflammation. In tissues other than kidney, glucocorticoids may be the primary endogenous activators of the MR [52]. A recent study showed rapid nuclear translocation (activation) of the MR in neurons of locally inflamed DRG. In this LID model, mechanical pain behaviors were ameliorated by addition of the specific MR antagonist eplerenone to the zymosan/incomplete Freund’s adjuvant used to locally inflame the DRG [53]. Some of this effect may be due to direct effects on neurons, because eplerenone applied to small diameter cultured neurons in vitro could reverse some of the excitatory changes induced by DRG inflammation (Figure 3). In light of the study by Gu et al. in which GR agonists had opposite effects at later time points [49], it will be important to determine if MR antagonists still have anti-nociceptive effects at later time points. In a study using an NP model, infiltration of the nerve root with the GR agonist dexamethasone at the time of NP application could block development of mechanical pain behaviors. Interestingly, a similar effect could be obtained by infiltrating lidocaine, and no additional benefit was obtained by applying both drugs [54]. This is consistent with studies on the role of abnormal neuronal activity in initiating various inflammatory changes (see above), and with some clinical studies (see below).

The possible roles of the GR and MR at the level of the spinal cord have also been examined. MR and GR are both found in the dorsal horn, and in one study using the CCD model, intrathecal GR agonists and MR antagonists could synergistically reduce thermal and mechanical pain behaviors. This suggests the two receptors have opposing actions in the spinal cord [55], similar to what was found with the DRG [53]. The same group found that intrathecal MR antagonists reduced expression of pro-inflammatory cytokines in both dorsal horn and DRG, as well as reducing spinal cord microglia activation [56]. However, in a neuropathic pain model, intrathecal application of GR antagonists attenuated pain [57], so the role of spinal GR in pain transmission is still unclear. In another study, intrathecal injection of the clinically used steroid prednisolone throughout the 2 week period following DRG compression blocked mechanical and thermal pain induced by a CCD model [58]. Interpretation of this experiment is confounded by the fact that prednisolone can activate both MR and GR *in vitro*[51]. Further studies on the spinal roles of MR and GR are needed, given the conflicting results in previous studies.

Epidural injection of glucocorticoids has become a relatively common treatment for low back pain that does not respond to more conservative treatments. However, randomized clinical trials of efficacy of epidural steroid injections for various forms of low back pain have had mixed results [59,60]. A relatively common finding is that they are effective in the short term (usually on the order of a month) but not in the longer term (e.g. [61-63]). Another common finding is that some benefit is obtained from injection of steroids, but that it does not differ significantly from injection of local anesthetic alone (e.g. [64,65]; steroid injections almost always include a local anesthetic to avoid pain from the injection procedure). This finding mirrors the preclinical study by Tachihara et al. discussed above. Given the extensive studies on the role of inflammation in preclinical models of back pain, the lack of unambiguous clinical efficacy of epidural steroids raises some interesting issues for those involved in preclinical research:

**Figure 4:**
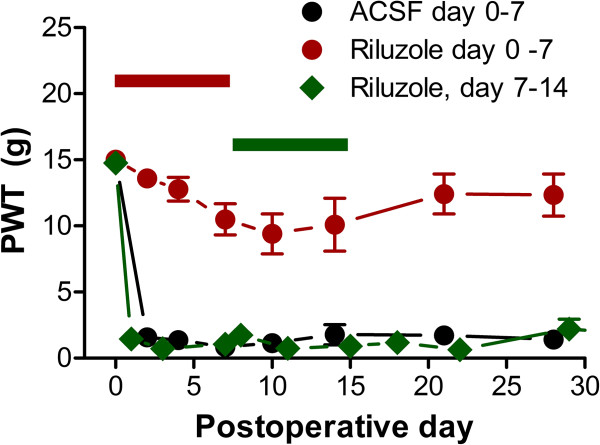
**A DRG level intervention is effective early after DRG inflammation, but not starting 1 week later.** Mechanical hypersensitivity (von Frey test) induced by DRG inflammation was increased by local inflammation of the DRG. This was greatly reduced by locally perfusing the DRG with riluzole, a blocker of persistent Na current, for 7 days starting at the time of inflammation (red; data adapted from reference [16]). DRG perfusion method was as shown in Figure 1. Perfusion with vehicle (ACSF; artificial cerebrospinal fluid, black symbols) was ineffective. However, riluzole perfusion starting 7 days after inflammation was ineffective (green symbols; unpublished data from our laboratory. N = 6 rats per group. No significant differences from the ACSF group were observed on any day by repeated measures ANOVA).

1. Might some forms of chronic back pain be due to an ongoing inflammatory process, which is not well modeled by current preclinical models? If stimuli leading to inflammation were ongoing, it might account for the failure of epidural steroid injections to provide long-lasting relief. Many studies of preclinical models of back pain are conducted at relatively short time points and may be more useful in understanding the initiation of the pain state rather than the chronic phase (which is however more relevant to patients with low back pain). It is not clear that any of the commonly used models could mimic ongoing inflammation.

2. Might some of the failures of epidural steroid injections in clinical trials be due to the fact that most of the steroids clinically used for this purpose also significantly activate the MR as well as the GR, and MR activation is pro-nociceptive at the DRG level? For example, the EC50 ratios for the GR affinity/MR affinity (where 1 = equal affinity and lower numbers indicate greater GR selectivity) as measured in cell systems expressing human receptors are greater than 1 for methylprednisolone and prednisolone; for triamcinolone the ratio is 0.1 [51]. The most selective among steroids commonly used for lumbar epidural injections seems to be betamethasone, which has a value of 0.02. However, even a highly GR-selective steroid might be more effective with the addition of an MR antagonist, if endogenous activators of the MR are present. In addition, the in vitro studies of MR and GR activation by various steroids examine transactivation of reporter genes; different EC50 values may apply to other mechanisms by which these receptors can interact to affect inflammation, such as transrepression (GR) or activation (MR) of the nuclear factor κ B (NFκB) pathway [66,67]. There is a need for more preclinical studies to examine clinically used steroids, delivered at the DRG level as occurs clinically.

3. Might some clinical failures of epidural steroid injections be due to spinal centralization? Nociceptive inputs to the spinal cord show this form of plasticity in which a long-lasting increase in the strength of the synaptic connections in the dorsal horn can be observed following intense, repeated, and sustained nociceptor activity [68,69]. If some forms of low back pain were due to central sensitization, this might account for some failures of steroid therapy directed at the DRG level. Some clinical studies have provided indirect evidence for central sensitization in some forms of chronic low back pain [70-73]. Preclinical low back pain models evoke changes in the spinal cord including glia activation and cytokine release, similar to those reported in studies of central sensitization in neuropathic pain models. In particular, some cytokines implicated in low back pain models have also been shown to play a role in central sensitization in other types of pain models. In the LID model, spontaneous activity of DRG neurons declines with time. In addition, the persistent Na channel blocker riluzole that effectively reduces mechanical pain when applied locally to the inflamed DRG if applied for the first 7 days, is ineffective when applied starting at day 7 (Figure 4). These results are consistent with the origin of pain moving from the DRG to the spinal cord with time. However, there are to date few preclinical studies directly examining long-term changes in spinal cord neurons and circuits in low back pain models, using methods similar to those used to study experimentally induced spinal cord sensitization.

All animal experimental procedures described herein were approved by the University of Cincinnati Institutional Animal Care and Use Committee (05-01-20-02) and were performed in accordance with the National Institutes of Health guidelines on animal care.

## Conclusions

Several preclinical models of low back pain have been developed, based on clinical conditions associated with back pain. Though differing in details and duration, these models share many similarities, including mechanical allodynia, increased excitability and spontaneous activity of sensory neurons, increased levels of pro-inflammatory cytokines, and other inflammatory changes. To date there are relatively few translational studies examining clinically used corticosteroids. Such studies might clarify the conditions under which such drugs might be effective.

## Abbreviations

CCD: Chronic compression of the DRG (back pain model); CFA: Complete Freund’s adjuvant; DRG: Dorsal root ganglion; GR: Glucocorticoid receptor; IL-1β: Interleukin 1β; LID: Local inflammation of the DRG (back pain model); MCP-1: Monocyte chemoattractant protein 1; MR: Mineralocorticoid receptor; NP: Nucleus pulposus; TNF-α: Tumor necrosis factor α.

## Competing interests

JAS, WX, and J-MZ have a patent pending application for the use of eplerenone in low back pain.

## Authors’ contributions

JAS drafted the manuscript and reviewed preclinical literature. WX helped write the manuscript and performed the experiments shown in Figures 2 and 4. FJB reviewed clinical literature and helped write the manuscript. J-MZ designed the review topic and helped write the manuscript. All authors read and approved the final manuscript.
